# Lead Service Lines and Infant Blood Lead Levels

**DOI:** 10.1001/jamanetworkopen.2025.50444

**Published:** 2025-12-17

**Authors:** Joanna Balza, Aprill Z. Dawson, David Nelson, Caitlin Kaeppler, Rachel Cusatis, Kathryn E. Flynn

**Affiliations:** 1 Department of Medicine, Medical College of Wisconsin, Milwaukee; 2 Department of Family Medicine, Medical College of Wisconsin, Milwaukee; 3 Department of Pediatric Medicine, Children’s Wisconsin, Milwaukee

## Abstract

This cross-sectional study investigates whether living in a residence with a lead service line is associated with blood lead levels among infants.

## Introduction

Lead can be deposited in water through lead service lines (LSLs).^1^ Among infants, particularly those who consume formula reconstituted with tap water, this may contribute to blood lead levels (BLLs).^2^ Approximately 65 000 residences in Milwaukee, Wisconsin, have LSLs.^3^ We examined the association between living in a Milwaukee residence with an LSL and BLL among infants, a potentially overlooked population at risk, as recommended lead screening does not start until 1 year of age.

## Methods

The Medical College of Wisconsin exempted this study from ethics review and informed consent because it did not involve direct participant contact. We adhered to the STROBE reporting guideline for cross-sectional studies. 

BLLs from 2018 to 2021 were obtained from the Wisconsin Department of Health Services. We included infants 12 months or younger who had venous BLL samples recorded as equal to a given value and excluded nonspecific values recorded as only less than or greater than a given value. The outcome variable was dichotomized at 3.5 µg/dL (to convert to µmol/L, multiply by 0.0483).

Covariates included age, sex, race and ethnicity, and Medicaid status. Season of testing was dichotomized into warmer and colder months based on reported seasonal BLL variation.^4^ Zip code was dichotomized as above or below 13.1% of residents at the federal poverty level.^5^ Age of residence was used as a proxy for risk of lead-based paint. Additional details are provided in the eMethods and eFigure in Supplement 1.

Data were analyzed from January 12, 2024, to October 27, 2025. Analyses using Stata, version 18 (StataCorp LLC) included bivariate analyses using χ^2^ tests (for categorical variables) and *t* tests (for continuous variables) and adjusted analyses using logistic regression with odds ratios (ORs). Sensitivity analyses used linear regression of the continuous BLL variable (log transformed) and logistic regression with alternative cut points. Two-sided *P* ≤ .05 was considered significant.

## Results

The study included 1210 infants (584 [48.3%] female, 626 [51.7%] male), 265 (21.9%) of whom had a BLL of 3.5 µg/dL or greater. LSL data were unavailable for 3 infants, race and ethnicity data for 94 infants, and age of residence for 18 infants; Over half of residences (653 of 1207 [54.1%]) had LSLs. Bivariate analysis yielded BLL differences between those living in a residence with an LSL vs without an LSL at the 3.5 µg/dL cut point (28% vs 15%). Complete details on sample characteristics are provided in Table 1.

**Table 1.** Study Sample Characteristics^a^

Characteristic	Total (N = 1210)	LSL at primary residence^b^		*P* value
		No (n = 554)	Yes (n = 653)	
BLL, µg/dL				
<3.5	945 (78.1)	472 (85.2)	470 (72.0)	<.001
≥3.5	265 (21.9)	82 (14.8)	183 (28.0)	
Age, mo				
Mean (SD)	11.28 (1.42)	11.23 (1.41)	11.32 (1.42)	.26
0-6	23 (1.9)	9 (1.6)	14 (2.1)	NA
7-12	1187 (98.1)	545 (98.4)	639 (97.9)	NA
Sex				
Female	584 (48.3)	268 (48.4)	313 (47.9)	.88
Male	626 (51.7)	286 (51.6)	340 (52.1)	
Race and ethnicity^c^				
Hispanic or Latino	194 (17.4)	63 (12.2)	131 (22.0)	<.001
Non-Hispanic Asian	153 (13.7)	82 (15.9)	71 (11.9)	
Non-Hispanic Black	544 (48.7)	263 (50.9)	278 (46.6)	
Non-Hispanic White	198 (17.7)	100 (19.3)	98 (16.4)	
Non-Hispanic other^d^	27 (2.4)	9 (1.7)	18 (3.0)	
Medicaid recipient				
No	216 (17.8)	102 (18.4)	114 (17.5)	.67
Yes	994 (82.2)	452 (81.6)	539 (82.5)	
Poverty by zip code^e^				
Below	275 (22.7)	200 (36.1)	75 (11.5)	<.001
Above	935 (77.3)	354 (63.9)	578 (88.5)	
Season of testing				
Colder months	510 (42.2)	226 (40.8)	283 (43.3)	.37
Warmer months	700 (57.8)	328 (59.2)	370 (56.7)	
Age of residence at time of testing, mean (SD), y^f^	86.70 (29.74)	63.70 (24.83)	105.75 (17.49)	<.001

Abbreviations: BLL, blood lead level; LSL, lead service line; NA, not applicable.

SI conversion factor: To convert BLL to µmol/L, multiply by 0.0483.

^a^ Data are presented as No. (%) of infants unless indicated otherwise. Percentages may not sum to 100 owing to rounding.

^b^ Data were unavailable for 3 infants.

^c^ These data were collected owing to previously reported associations between race and ethnicity and BLLs. Data were unavailable for 94 infants.

^d^ Includes American Indian or Alaska Native, multiracial, and other race or ethnicity.

^e^ Zip code–level poverty is below or above 13.1% of residents at the federal poverty level.

^f^ Data were unavailable for 18 infants.

After adjusting for covariates, LSLs were associated with a BLL of 3.5 µg/dL or greater (OR, 1.60 [95% CI, 1.04-2.46]; *P* = .03) (Table 2). Sensitivity analyses yielded similar results after adjusted logistic regression at a cut point of 1 µg/dL or less (OR, 1.72 [95% CI, 1.21-2.44]; *P* = .002), adjusted logistic regression at a cut point of 5.0 µg/dL or greater (OR, 2.26 [95% CI, 1.23-4.16]; *P* = .008), and linear regression of the continuous BLLs (0.13 [95% CI, 0.04-0.22]; *P* = .004).

**Table 2.** Adjusted Logistic Regression Results (n = 1101)

Variable	BLL ≥3.5 µg/dL, OR (95% CI)	*P* value
LSL at primary residence		
No	1 [Reference]	NA
Yes	1.60 (1.04-2.46)	.03
Age^a^	1.28 (1.12-1.46)	<.001
Sex		
Female	1 [Reference]	NA
Male	1.07 (0.80-1.45)	.64
Race and ethnicity^b^		
Hispanic or Latino	0.57 (0.33-0.98)	.04
Non-Hispanic Asian	0.71 (0.40-1.27)	.25
Non-Hispanic Black or African American	0.88 (0.53-1.44)	.61
Non-Hispanic White	1 [Reference]	NA
Non-Hispanic other^c^	0.47 (0.15-1.50)	.20
Medicaid recipient		
No	1 [Reference]	NA
Yes	1.08 (0.67-1.73)	.76
Poverty by zip code^d^		
Below	1 [Reference]	NA
Above	0.83 (0.53-1.30)	.42
Season of testing		
Colder months	1 [Reference]	NA
Warmer months	0.90 (0.67-1.22)	.50
Age of residence at time of testing^a^	1.01 (1.00-1.02)	.02

Abbreviations: BLL, blood lead level; LSL, lead service line; NA, not applicable; OR, odds ratio.

SI conversion factor: To convert BLL to µmol/L, multiply by 0.0483.

^a^ Continuous variable; OR pertains to increase per unit.

^b^ These data were collected owing to previously reported associations between race and ethnicity and BLLs.

^c^ Includes American Indian or Alaska Native, multiracial, and other race or ethnicity.

^d^ Zip code–level poverty is below or above 13.1% of residents at the federal poverty level.

## Discussion

These findings suggest that residing in a home with an LSL was associated with increased odds of infants having a BLL of 3.5 µg/dL or greater. Given the multiple sources of exposure, elucidating the impact of LSLs is useful for prioritizing prevention efforts. BLL testing is recommended starting at age 12 months, but lead has a half-life of only 25 to 36 days; therefore, traditional surveillance may not detect the impact of water lead on infants.

Study limitations include the following unavailable variables: length of time infant resided at residence; where the majority of time was spent; if the infant was fed formula; if tap, bottled, or filtered water was used; if pipes were flushed or other actions were taken to reduce water lead; and other potential sources of lead. Additionally, some residences may have had LSLs removed during the data collection period. Further studies are needed to examine whether current filter distribution interventions are reaching at-risk populations. Additionally, targeted interventions such as distributing filters at prenatal visits may be useful.

## Notes

Supplement 1. **eMethods.** Details on the Dataset, Variables, and Analysis Methodology **eFigure.** Selection of Participants for the Analytic Sample
eReferences

Supplement 2.
Data Sharing Statement
