# Supplementary material for: Lead Service Lines and Infant Blood Lead Levels
Source: JAMA Netw Open. 2025 Dec 17;8(12):e2550444. doi: 10.1001/jamanetworkopen.2025.50444 (PMC12712725; doi:10.1001/jamanetworkopen.2025.50444)
Supplement: Supplement 1. — eMethods. Details on the Dataset, Variables, and Analysis Methodology eFigure. Selection of Participants for the Analytic Sample eReferences [file jamanetwopen-e2550444-s001.pdf]

Supplemental Online Content

Balza J, Dawson AZ, Nelson D, Kaeppler C, Cusatis R, Flynn KE. Lead service lines and infant blood lead levels. *JAMA Netw Open*. 2025;8(12):e2550444. doi:10.1001/jamanetworkopen.2025.50444

eMethods. Details on the Dataset, Variables, and Analysis Methodology
eFigure. Selection of Participants for the Analytic Sample
eReferences

This supplemental material is provided by the authors to give readers additional detail about the methodology of the study.

© 2025 Balza J et al. *JAMA Network Open*.

eMethods. Details on the Dataset, Variables, and Analysis Methodology

This study was approved by the institutional review board at the Medical College of Wisconsin. This study was guided by the STROBE guidelines checklist for cross-sectional studies, to the extent they were applicable in the format of a brief research letter (e.g. no abstract included).

Dataset and Study Subsample

The outcome variable, blood lead level (BLL), used blood lead test results for children in the city of Milwaukee obtained thought the State of Wisconsin Department of Health Services (DHS). The data set included BLL results for Milwaukee children tested between 2018-2021, as well as a unique identification number, race, ethnicity, sex, age of child at testing (in months), Medicaid Status, codes indicating if the draw was venous or capillary, codes signifying if the BLL value was equal to, less than, or greater than the listed value, and primary address of residence. For inclusion in this analysis the data set was limited to children 12 months and younger, to assess the impact on those who may experience a strong impact of water lead (especially if consuming infant formula prepared with tap water). The data were limited to children with venous draw samples, as they are known to be more accurate than capillary samples.[1,2] BLL results in the data set were labeled as 'equal to', 'less than' or 'greater than' a certain value. For our study, we limited the data to exact values, or those that were labeled 'equal to'. In other words, less specific results that were coded as 'greater than' or 'less than' a certain value, such as <5 µg/dl (which could mean any value between 0 and 4.9) were not included. If a child had more than one qualifying BLL value, the first venous/exact value was used. eFigure 1 represents the process of participant selection for analytic sample.

Dependent Variable

The primary dependent variable was BLL. Examination of the BLL data showed that values trended strongly towards integers rather than decimals, indicating lead level sensitivity limitations in some laboratories, despite having only used values categorized as 'equal to' in the data set. Therefore, to enhance clinical relevance, the variable was dichotomized and analyzed at a 3.5 µg/dl cut point (the current Centers for Disease Control and Preventions' blood reference value), a procedure used in previous literature.

Primary Independent Variable

The primary independent variable, presence of a lead service line to the listed primary residence of the child, was found using public lead service line data as of 2022 from the Milwaukee Water Works[3] for each address listed as a child's primary residence in the DHS data. Lead service line data were available for 99.8% of addresses. A random sample of 10% of the values were independently found a second time and compared to the initial values for quality control, which showed complete accuracy.

Covariates

The selection of covariates was based on literature review incorporating demographic factors (race/ethnicity, age, sex), socioeconomic variables (Medicaid status and zip code), testing variables (season of testing), and a housing specific variable (age of housing). If not otherwise noted, data were from the DHS dataset. Demographic factors included race and ethnicity, which were reported to DHS by the reporting lab or provider, presumably as reported in the patient's electronic health record collected by the lab or provider. Reported ethnicity by DHS included: Hispanic or Latino, Non-Hispanic or Latino, and Unknown. DHS reported race included: American Indian or Alaska Native, Asian, Black or African American, Multiracial, Unknown, and White. Using Stata, we recoded into the following categories: Hispanic or Latino, Non-Hispanic Asian, Non-Hispanic Black, Non-Hispanic Other or Multiracial, and Non-Hispanic White). Sex was reported as male or female, and Age was reported in months as a continuous variable. Month of testing was dichotomized into warmer months (April-September) and cooler months (October-March), based on prior literature reporting seasonal variations in BLL.[4–6] Zip code was dichotomized as above or below 13.1% of residents at the federal poverty level (median Milwaukee County zip code poverty, 2017-2021, as found in the American Community Survey[7] and reported by Health Compass Milwaukee in January 2024).[8] Age of housing at time of testing was used as a proxy for potential presence of lead paint and housing condition; it was calculated by subtracting the year the housing was constructed (data from the City of Milwaukee City Clerk's records[9]) from the year of testing.

© 2025 Balza J et al. *JAMA Network Open*.

Analysis

All analyses were performed using Stata SE 18. First, a bivariate analysis of the covariates was performed using t-tests for continuous variables (age of child, age of house) and chi square tests for categorical variables (BLL $<$ or ≥ 3.5, sex, race/ethnicity, Medicaid, zip code, season of testing). Next, an adjusted logistic regression model was used to determine the association between a lead service line and BLL at the ≥ 3.5 ug/dL cutpoint. Finally, sensitivity analyses were performed using logistic regression at the ≤ 1 ug/dL and ≥ 5.0 ug/dL cutpoints, and then with a linear regression using the continuous blood lead variable transformed as log(BLL variable+1) to address the distribution and accommodate zero values. A p-value ≤ 0.05 was considered statistically significant.

eFigure. Selection of Participants for the Analytic Sample

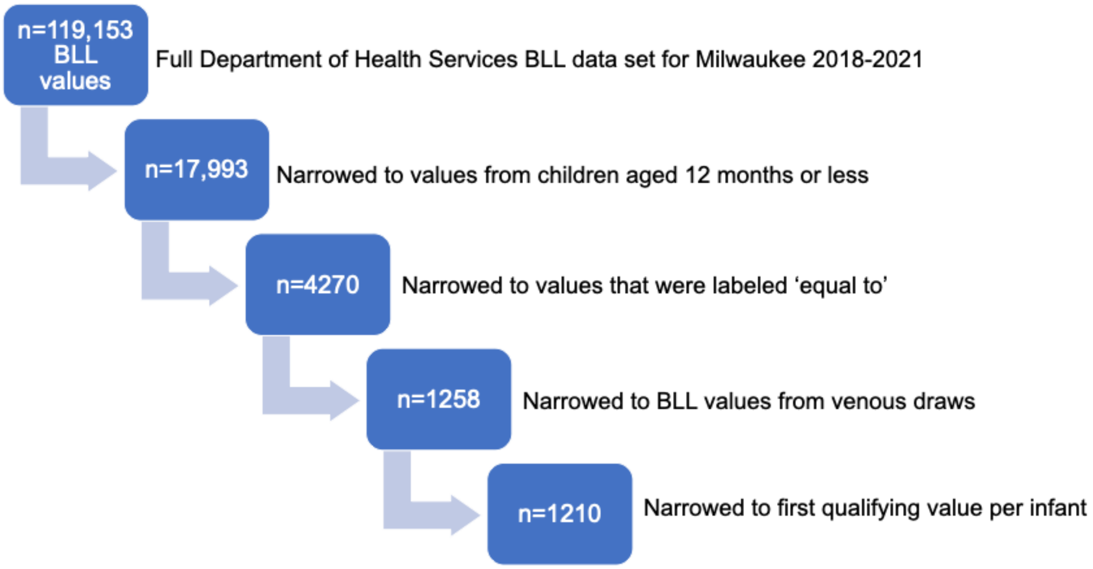

© 2025 Balza J et al. *JAMA Network Open*.

eReferences

1. Wang A, Rezania Z, Haugen KMB, Baertlein L, Yendell SJ. Screening for Elevated Blood Lead Levels: False-Positive Rates of Tests on Capillary Samples, Minnesota, 2011-2017. *Journal of Public Health Management and Practice*. 2019;25(1). doi:10.1097/PHH.0000000000000879

2. Anderson MK, Amrich M, Decker KL, Mervis CA. Using State Lead Poisoning Surveillance System Data to Assess False Positive Results of Capillary Testing. *Matern Child Health J*. 2007;11(6). doi:10.1007/s10995-007-0196-1

3. City of Milwaukee MWW. Lead Service Line Records. https://city.milwaukee.gov/water/WaterQuality/LeadandWater/Lead-Service-Line-Records

4. Ngueta G, Prévost M, Deshommes E, Abdous B, Gauvin D, Levallois P. Exposure of young children to household water lead in the Montreal area (Canada): The potential influence of winter-to-summer changes in water lead levels on children's blood lead concentration. *Environ Int*. 2014;73:57-65. doi:10.1016/j.envint.2014.07.005

5. Haley VB, Talbot TO. Seasonality and trend in blood lead levels of New York State children. *BMC Pediatr*. 2004;4(1):8. doi:10.1186/1471-2431-4-8

6. Laidlaw MAS, Mielke HW, Filippelli GM, Johnson DL, Gonzales CR. Seasonality and Children's Blood Lead Levels: Developing a Predictive Model Using Climatic Variables and Blood Lead Data from Indianapolis, Indiana, Syracuse, New York, and New Orleans, Louisiana (USA). *Environ Health Perspect*. 2005;113(6):793-800. doi:10.1289/ehp.7759

7. United States Census Bureau. American Community Survey (ACS). https://www.census.gov/programs-surveys/acs

8. Health Compass Milwaukee. Families Living Below Federal Poverty Level. https://healthcompassmilwaukee.org/topics/POV?tab=table

9. City of Milwaukee. City Clerk's Office: Property Records. https://city.milwaukee.gov/cityclerk/PublicRecords/Property-Records

© 2025 Balza J et al. *JAMA Network Open*.
