# Supplementary material for: Lead Service Lines and Infant Blood Lead Levels
Source: JAMA Netw Open. 2025 Dec 17;8(12):e2550444. doi: 10.1001/jamanetworkopen.2025.50444 (PMC12712725; doi:10.1001/jamanetworkopen.2025.50444)
Supplement: Supplement 2. — Data Sharing Statement [file jamanetwopen-e2550444-s002.pdf]

Data Sharing Statement

Balza. Lead Service Lines and Infant Blood Lead Levels. *JAMA Netw Open*. Published December 17, 2025. doi:10.1001/jamanetworkopen.2025.50444

Data
Data available: No

Additional Information
Explanation for why data not available: We accessed this data through a data use agreement with the Wisconsin Department of Health Services, as well as publicly available data from Milwaukee Water Works. While we are not authorized to release the data according to our data use agreement with WI DHS, others can also access the data by establishing their own data request to WI DHS.
